# Yellow Fever in New Orleans

**Published:** 1853-10

**Authors:** 


*Yellow Fever in New Orleans.*—About the 26th of May last, the first case of yellow fever entered the Charity Hospital, and after death black vomit was found in the stomach. The first fever cases originated among the shipping along the Levee, in the Fourth District, from which point it extended rapidly through the adjacent portion of the town. A large population of unacclimated persons, living in wooden huts, with floors and timbers soaked in water, and half decayed, were seized with the disease in the most malignant form. For some time previously rain had fallen almost daily, and this added to a hot, burning sun, seemed to give strength to the poison, and lent intensity to the disease. The streets in this vicinity, for the most part, were unpaved, or planked, and the culverts, gutters, etc., were filled with water, saturated with filth and decaying vegetable and animal matter. The crowded state of these huts and low wooden tenements, with their floors steeped in mud and water, is admirably calculated to generate and propagate the germ of a disease which had already been sown in their midst.

The habits of these people,(being chiefly Irish and German laborers,) notoriously negligent and filthy, and utterly indifferent to all those precautionary measures which a limited knowledge of the laws of hygiene should suggest, served only to add fuel to the conflagration which was destined to extend its ravages to every portion of our devoted city. Hence, for some time, the yellow fever confined its work of death within particular localities,—but by and by gaining strength by what it fed upon, it began to travel to other and more distant points,—to extend its arms, so to speak, in every direction, until it grasped the Four Districts within its deadly embrace. For some time the hope was entertained that those who paid proper regard to personal comfort and cleanliness—who dwelt in high, airy, and well-ventilated apartments might escape the disease ; but this proved a delusion,— it soon became apparent that as heretofore, the epidemic fever was no respecter of persons,—the master was stricken down with the servant—the mistress with the maid—the proud and wealthy were brought to a level with the humble and needy. All who had not passed through some one of our epidemic seasons were exposed to attacks from the disease. As has been already mentioned, the fever made its appearance in the latter part of May, at least one month and a half earlier than usual, and from the first case up to the present, it steadily increased almost daily, until the mortality per diem exceeded that produced by any epidemic known in the annals of our sanitary history. In recording the fearful ravages of the present epidemic we must not forget that we have remained exempt from any such visitation since 1847, and during this time an immense population of unacclimated persons, both from Europe and the north-western part of our own country, have been accumulating in our city. The number of unacclimated persons in the city, at the breaking out of the epidemic has been estimated at 30,000 souls; but many of these, it is fair to suppose, have left the city to escape the disease.

The type of the epidemic differs but little from that to which we have been subject in former years; and the belief that persons had died of the disease in six and eight hours from the moment of seizure, can readily be explained by a better knowledge of the antecedent history of the case; for on inquiry it would generally be found that such individuals have had slight fever and other symptoms of the epidemic for two or three days previously to taking their bed and calling in medical aid. This surmise gains additional stregth from the fact that the attack, in many instances, has been so insiduous and destitute of alarming symptoms, that it is with difficulty such persons could be persuaded—could be prevailed upon to submit to the usual restrictive treatment.

It is not strange therefore that such cases, which had been neglected for two or three days, in the early and curable stage of attack, should terminate in fatal black vomit, in a few hours after the physician is summoned to the bedside of his patient. So much for the apparent malignancy of the present epidemic. In making the foregoing explanation, we aim not to deny the existence of an occasional case of extreme severity; so severe indeed as to terminate in death in a few hours, in spite of the best efforts of the most skillful physician and the most careful nursing.

In some instances the system seems so thoroughly saturated with the poison of the disease, from the very moment of seizure, that no system of medication, as yet suggested, seems able to cope with and stay the fatal tendency of the fever. Every medical man who has had much experience in the disease, must remember occasional instances of this kind.

The disease this season, though essentially the same in many of its most prominent features, exacts perhaps, on the part of physician and nurse, more care, diligence and precaution, to terminate favorably, than usual in our epidemics. The slightest imprudence, either in diet, exposure, or excitement of any kind, is almost certain to superinduce a relapse—from which state it is usually very difficult to extricate the patient. Hence, the great mortality among those who are not only ignorant of the peculiarities of the disease; but who are also unable, and in some instances unwilling to pay for the requisite medical aid and attendance.

We refer to our table below, furnished by Dr. Simonds, the active Secretary of the Board of Health, for a full account of the deaths and other particulars which have occurred since the epidemic broke out. By this, it will be seen that yellow fever has done terrible execution among our unacclimated population; has produced a mortality unparalleled in the history of our ill-fated city. Even while penning these lines, the fever is sweeping off over *two hundred per diem*—and from present appearances, it is likely to continue its fearful ravages for, perhaps, weeks to come.

Our quondam associate, Dr. Fenner will, in due time, give us a full and detailed history of this epidemic, as he did that of 1847, when the disease shall have run its course and done its work of death.

On the next page, we give the mortality produced by the epidemic, in the city of New orleans, from the 28th of May up to the 26th of August, inclusive, for 1853.

### THE EPIDEMIC.

*Total number of Deaths by Yellow Fever and Other Diseases, from May 28 till date.*

| Week Ending. | Total. | Yellow Fever. | Other Disea's | Not Stated. |
|---|---|---|---|---|
| May 28 | 140— 110 | 1— 1 | 139— 139 | .. |
| June 4 | 157 | 1 | 156 | .. |
| ...... 11 | 154 | 4 | 150 | .. |
| ...... 18 | 147 | 7 | 140 | .. |
| ...... 25 | 167— 625 | 9— 21 | 158— 604 | .. |
| July 2 | 177 | 25 | 152 | .. |
| ...... 9 | 188 | 59 | 129 | .. |
| ...... 16 | 344 | 204 | 140 | .. |
| ...... 23 | 617 | 435 | 182 | .. |
| ...... 31 | 884—2210 | 704—1427 | 138— 741 | 42 |
| August 1 | 142 | 106 | 25 | 11 |
| ...... 2 | 135 | 115 | 14 | 6 |
| ...... 3 | 146 | 124 | 17 | 9 |
| ...... 4 | 166 | 135 | 15 | 10 |
| ...... 5 | 150 | 128 | 9 | 13 |
| ...... 6 | 238 | 194 | 30 | 14 |
| ...... 7 | 209—1186 | 165— 967 | 40— 150 | 4— 60 |
| ...... 8 | 219 | 187 | 23 | 9 |
| ...... 9 | 201 | 166 | 21 | 14 |
| ...... 10 | 230 | 193 | 33 | 4 |
| ...... 11 | 233 | 192 | 13 | 18 |
| ...... 12 | 207 | 180 | 25 | 2 |
| ...... 13 | 214 | 179 | 2[illegible] | 13 |
| ...... 14 | 232—1526 | 191—1288 | 26— 163 | 16— 75 |
| ...... 15 | 217 | 187 | 24 | 6 |
| ...... 16 | 193 | 163 | 19 | 11 |
| ...... 17 | 219 | 191 | 21 | 7 |
| ...... 18 | 219 | 188 | 22 | 9 |
| ...... 19 | 234 | 203 | 15 | 16 |
| ...... 20 | 224 | 184 | 29 | 11 |
| ...... 21 | 269—1575 | 230—1346 | 24— 154 | 15— 75 |
| ...... 22 | 283 | 239 | 29 | 15 |
| ...... 23 | 258 | 220 | 24 | 14 |
| ...... 24 | 222 | 188 | 23 | 11 |
| ...... 25 | 218 | 186 | 19 | 13 |
| ...... 26 | 193—1074 | 151— 884 | 29— 124 | 13— 66 |
| Total | 8336 | 5934 | 2075 | 327 |

N.B. The returns from St. Patrick's Cemetery since the 31st July, not having been duly made, cannot be relied on, except for two weeks, when the books were resorted to by the Secretary, to enable him to make a weekly report.—*N. O. Med. and Surg. Jour.*

